# Mixed Mycobacterium Avium-Intracellulare and Serratia Marcescens Cellulitis of the Breast in an HIV-Negative Patient with Breast Cancer: A Case Report

**DOI:** 10.7759/cureus.634

**Published:** 2016-06-09

**Authors:** Andreas Kyvernitakis, Jacques Azzi, Dimitrios P Kontoyiannis

**Affiliations:** 1 Infectious Diseases, University of Texas MD Anderson Cancer Center; 2 Internal Medicine, Staten Island University Hospital

**Keywords:** nontuberculous mycobacterium, breast cancer, skin infection

## Abstract

*Mycobacterium avium-intracellulare *(MAI) causes pulmonary infection in patients with chronic lung diseases or severe T-cell deficiency. Cutaneous manifestations caused by MAI are rare and the few cases reported describe mostly patients with hematologic malignancies who were treated with highly immunosuppressive agents. Herein, we report a case of a breast cancer survivor who developed chronic breast cellulitis due to MAI, following localized breast cancer treatment.

## Introduction

*Mycobacterium avium-intracellulare (*MAI), the most common nontuberculous mycobacterium in the world, is an opportunistic pathogen that is notorious for causing pulmonary infections in patients with chronic lung disease and disseminated disease in patients with acquired immune deficiency syndrome [1-2]. Skin and soft tissue infection (SSTI) secondary to MAI is a rare entity in the absence of mycobacterial bloodstream infection (BSI). To our knowledge, the following report is the first description of MAI-related SSTI in a patient with a solid tumor [1, 3-6]. MAI soft tissue manifestations in patients with solid tumors have not been described before. To our knowledge, this is the first patient case reported of MAI soft tissue infection in a solid tumor. Informed consent was obtained from the patient for this study.

## Case presentation

This is a 71-year-old female who was recently diagnosed with a 1.2 cm left breast ductal carcinoma in situ (estrogen receptor weakly positive/progesterone receptor negative). She underwent a left segmental mastectomy with sentinel lymph node biopsy followed by adjuvant radiation therapy and placement of a catheter evacuation device. On her three-month follow-up visit, the patient complained of left breast tenderness, erythema, and a foul-smelling nipple discharge. Her symptoms had been ongoing for two weeks during which she also experienced intermittent low grade fevers and malaise. Physical examination was notable for a markedly tender left breast with associated erythema, induration (11 cm x 9 cm), and skin thickening overlying the site of radiation. Ultrasound of the breast revealed an underlying subcutaneous seroma measuring 4 cm × 8 cm × 5.2 cm. Subsequent aspiration of the cavity recovered 40 ml of purulent fluid from which *Serratia marcescens* was eventually isolated.​Of interest, the patient had an *S. marcescens *urinary tract infection six months ago. The patient was started on a seven-day regimen of ciprofloxacin after which she continued to experience purulent nipple discharge and diffuse breast erythema. Her course was further complicated by the development of a diffuse pruritic maculopapular rash that prompted the discontinuation of ciprofloxacin.

**Figure 1:**
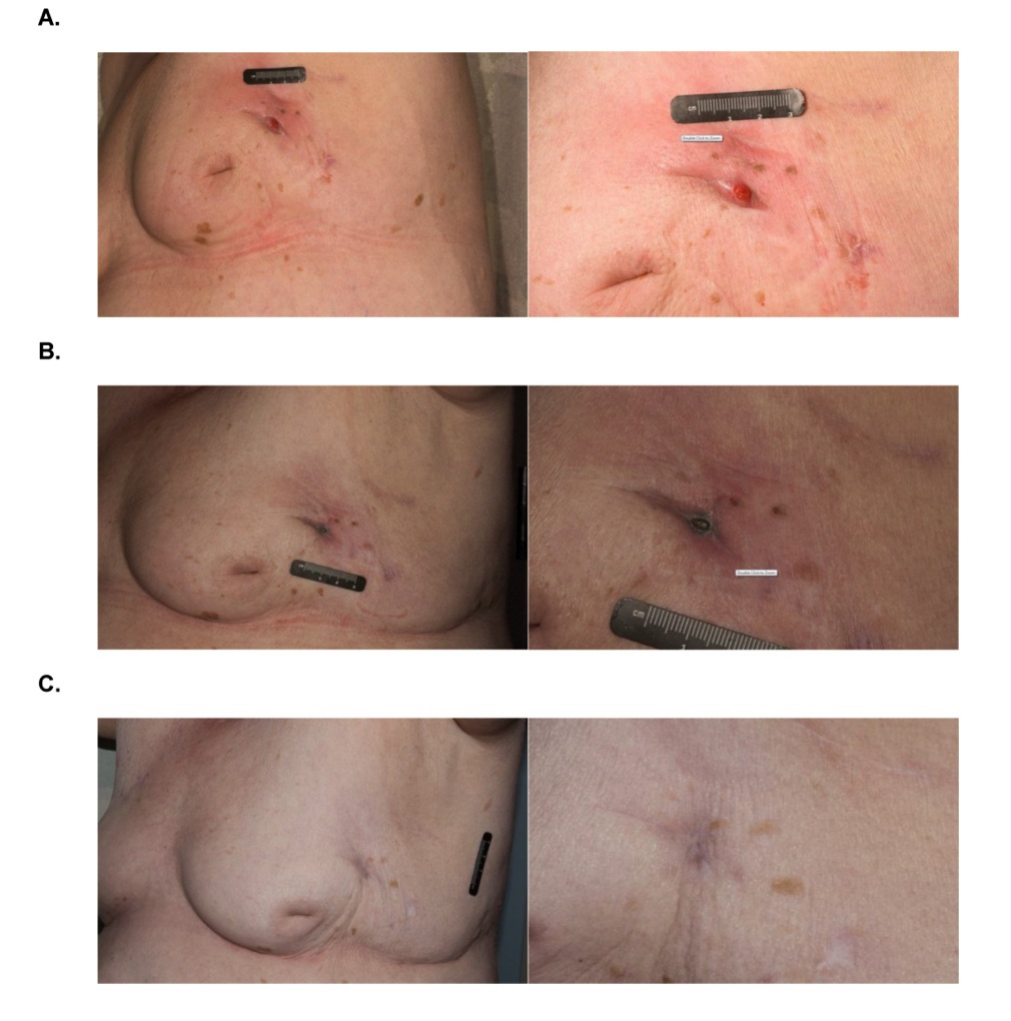
Breast seroma infected with Mycobacterium avium-intracellulare and Serratia marcescens Lateral and close-up views of the skin manifestations attributed to *Mycobacterium avium-intracellulare* and *Serratia marcescens *at: A. Four weeks of treatment. B. 10 weeks of treatment. C. 12 months after the end of treatment.

Four weeks after the aspiration, cultures also grew MAI. At that point, there was a modest improvement of the breast cellulitis, but persistent drainage of purulent fluid (Fig. 1A). Human immunodeficiency virus (HIV) serology results were negative. She was placed on an oral combination regimen, consisted of rifabutin (300 mg/day), clarithromycin (500 mg twice/day) and doxycycline (100 mg twice/day). Doxycycline, although an unconventional agent for the treatment of *S. marcescens,* was used instead of quinolones due to the patient’s recent history of hypersensitivity to ciprofloxacin, as it has in vitro activity against *S. marcescens *[7]. The patient’s seroma was aspirated again, draining 10 ml of yellow fluid that was sent for cultures and returned negative (Fig. 1B). In addition to rifabutin and clarithromycin, the patient was placed on intravenous ertapenem (1000 mg/day), followed by a course of oral cefixime (400 mg/day), and she received local wound care with topical silver nitrate, leading to a complete resolution of the erythema and drainage. Follow-up for the next 12 months showed progressive healing, and follow-up cultures did not grow MAI or *Serratia* (Fig. 1C).

## Discussion

SSTIs secondary to MAI infection are thought to occur via hematogenous seeding in the context of an underlying trauma. Some of the well-established cutaneous manifestations include scaling papules, subcutaneous/ulcerative nodules, granulomatous plaques, and abscesses [3, 8]. Presumptively, in our patient, the recent breast surgery and radioadjuvant therapy served as the nidus for seeding *S. marcescens* and MAI. The few reports existing in the literature of cutaneous MAI infection in non-HIV infected cancer patients describe mostly leukemia patients who were treated with highly immunosuppressive agents [1, 6].

A combination of surgical debridement and a three-drug regimen with macrolides (clarithromycin or azithromycin), ethambutol, and a rifamycin (rifampin or rifabutin) may be extrapolated from studies of HIV-infected patients. [2, 9-10].

## Conclusions

While an optimal treatment for an extra-pulmonary MAI infection isolated to the soft tissue has not yet been defined, a combination of surgical debridement and a three-drug regimen with macrolides, ethambutol, and rifamycin may provide a successful outcome and should be considered.
